# Supplementary material for: COVID-19 Vaccine Acceptability and Adherence to Preventive Measures in Somalia: Results of an Online Survey
Source: Vaccines (Basel). 2021 May 21;9(6):543. doi: 10.3390/vaccines9060543 (PMC8224389; doi:10.3390/vaccines9060543)
Supplement: Supplementary file 1 [file vaccines-09-00543-s001.zip › File S1.pdf]

SOMALIA THIRD ROUND / FADLAN RIIX HALKAN

Socio-demographic information/ Macluumaadka Qofka

Q: Did you fill the previous online questionnaire for COVID-19 in July 2020?/Maka qeyb qaadatay Sahmintii koowaad ee Covid-19 ee July 2020 ? (*)

Type: choice

A: one of the following:

yes	=>	*Yes, I filled the previous questionnaire/Haa waan buuxiyay Sahmintii koowaad*
no	=>	*No, this is my first time to fill this questionnaire/Maya waa markii koowaad ee aan buuxiyo sahmintani*

Q: In which region of Somalia do you live?/Halkeed uga nooshahay soomaaliya ? (*)

Type: choice

A: one of the following:

Benadir	=>	*Benadir/banaadir*
Galmudug	=>	*Galmudug/Galmudug*
Hirshabelle	=>	*Hirshabelle/Hirshabeelle*
Jubaland	=>	*Jubaland/Jubaland*
Somaliland	=>	*Somaliland/Soomaliland*
Southwest	=>	*Southwest/Koonfur galbeed*
Puntland	=>	*Puntland/Buntland*

Q: Age/Da'da (*)

Type: number

A: number (min: 18 / max: 110 / step: 1)

Q: Sex/ Jinsiga (*)

Type: choice

A: one of the following:

male	=>	*Male/Lab*
female	=>	*Female/Dhedig*

Q: Nationality /Jinsiyadda (*)

Type: choice

A: one of the following:

local	=>	*I am a local/Waxaan ahay maxalli/muwaadin*
foreigner	=>	*I am a foreigner/Waxaan ahay ajnabi*

Q: Religion/Diinta (*)

Type: choice

A: one of the following:

christian	=>	*Christian/Masiixi*
muslim	=>	*Muslim/Muslim*
other	=>	*Other/Wax kale*
none	=>	*None/Midna*

Q: Highest educational level/Aqoonta waxbarasho (*)

Type: choice

A: one of the following:

primary	=>	*Primary/Dugsi hoose/dhexe*
Secondary	=>	*Secondary/Dugsi sare*
University Undergraduate degree holder	=>	*University Undergraduate degree holder/Jaamici shahaadada koowaad*
University Postgraduate degree holder	=>	*University Postgraduate degree holder/Jaamici shahaadada sare*

Q: Marital status/Xaaladda Guurka (*)

Type: choice

A: one of the following:

single	=>	*Single/Doob*
legally_married	=>	*Legally married/sharciyan xaasle*
cohabitation	=>	*Cohabitation/Qof la nool*
divorced	=>	*Divorced/Garoob*
widow_widower	=>	*Widow/widower/Carmali*

Q: Who do you currently live with? (many answers possible)Yaad hadda la nooshahay? (Jawaabo badan ayaad bixin kartaa) (*)

Type: choice_multiple

A: multiple answers possible:

My parent(s)	=>	*My parent(s)/Waalidiinteyda*
My spouse/partner	=>	*My spouse/partner/Xaaskeyga ama saaxiib*
My child(ren)	=>	*My child(ren)/Caruurteyda*
My sibling(s) or other relative(s)	=>	*My sibling(s) or other relative(s)/Walaalahey ama qaraabo kale*
Friends	=>	*Friends/saaxiibbadey*
I live alone	=>	*I live alone/Kalideey ayaan noolahay*

Q: Do you live in:/Ma waxaad ku nooshahay: (*)

Type: choice

A: one of the following:

rural_area	=>	*a rural area/village/baadiye/tuulo*
suburb	=>	*suburb/Nawaaxiga magaalo*
provincial_town	=>	*provincial town/Degmo goboleed*

city	=>	*city/Magaalo*

Professional life during the coronavirus epidemic/ Nolashaadii shaqda intii uu jiray safmarka coronavirus

Q: What do you do for a living?/Maxaad ka shaqayn jirtay? (*)

Type: choice

A: one of the following:

student	=>	*Student/Arday*
jobless	=>	*Jobless/Shaqo la'aan*
self_employed	=>	*Self-employed/Anaa iskay u shaqeysan jiray*
company	=>	*Work for a person, institution or company/Waxaan u shaqeyn jiray qof, hay`ad ama shirkad*
government	=>	*Work for the government/ Dowladda ayaan u shaqeyn jiray*

Q: Are you a healthcare worker or a student working in the health sector?/Miyaad tahay shaqaale caafimaad ama arday ka shaqeynaya qeyb caafimaad? (*)

Type: choice

A: one of the following:

yes	=>	*Yes /Haa*
no	=>	*No /Maya*

Q: How many days per week do you usually go to school or work?/Meeqa maalin ayaad badanaa toddobaadkii tagtaa iskuulka ama shaqada? (*)

Type: number

A: number (min: n/a / max: 7 / step: n/a)

Q: How many days did you (physically) go to school or work last week?/Meeqa maalin ayaad tagtay iskuulka ama shaqadda toddobaadkii la soo dhaafay? (*)

Type: number

A: number (min: n/a / max: 7 / step: n/a)

Q: Are you working from home today?/Maanta ma guriga ayaad shaqadaadii ku qabanaysaa? (*)

Type: choice

A: one of the following:

yes	=>	*Yes /Haa*
no	=>	*No /Maya*
not_applicable	=>	*Not applicable (if jobless or student)/ima khusayso (qof ah shaqo la'aan ama arday)*

Q: Why are you not working from home?/maxaad shaqada guriga ugu dhex qaban wayday? (*)

Type: choice

A: one of the following:

not_possible	=>	*It is not possible with my job /Shaqadeydu kuma habboona*
not_allowed	=>	*It is possible, but is not allowed by my employer/Wey ku habboon tahay laakiin madaxdeydu ma oggola*
leave_house_make_money	=>	*I have to leave the house to make money to support my family/Waa in aan guriga ka baxo si aan lacag u soo shaqaysto oo aan qoyskeyga ku biilo*
home_not_working	=>	*I am at home but not working/ Guriga ayaan joogaa laakiin ma shaqaynayo*
no_risk_to_go_out	=>	*I don't think there is a risk to go out/U malayn maayo in khatar loo bixi waayaa ay jirto*
other	=>	*Other/wax kale*

Visible if

Q:	A:
Are you working from home today?/Maanta ma guriga ayaad shaqadaadii ku qabanaysaa?	- value => no

Personal preventive measures for coronavirus/Tallaabooyinka ka-hortagga shaqsiyeed ee coronavirus

Q: I wear a face mask when going outside/Waxaan xirtaa maaskiga wajiga marka aan dibadda u baxayo(*)

Type: choice

A: one of the following:

yes	=>	*Yes /Haa*
no	=>	*No /Maya*

Q: If yes, which kind of mask do you use? /Hadday haa tahay, maaska noocee ah ayaad isticmaashaa?(*)

Type: choice

A: one of the following:

disposable	=>	*Disposable mask/Maaskaro la tuuri karo*
reusable_cloth	=>	*Reusable cloth mask/Maaskaro dib loo isticmaali karo*
professional	=>	*Professional (strong, specialized) mask/Maaski Xirfadle (adag, khaas ah)*

Visible if

Q:	A:
I wear a face mask when going outside/Waxaan xirtaa maaskiga wajiga marka aan dibadda u baxayo	- value => yes

Q: If yes, when/where do you wear face masks?/Hadday haa tahay, goorma / xagee ayaad urxirtaa waji xidhka wejiga?(*)

Type: choice_multiple

A: multiple answers possible:

sometimes	=>	*Sometimes when I go out/Mararka qaar markaan baxo*
all_the_time	=>	*Every time I go out/Markasta oo aan baxo*
home	=>	*At home/Guriga*
work	=>	*At work/shaqada*

Visible if

Q:	A:
I wear a face mask when going outside/Waxaan xirtaa maaskiga wajiga marka aan dibadda u baxayo	- value => yes

Q: If no, why don't you use face masks?/Hadday maya tahay, maxaad u adeegsan weji xidhka? (*)

Type: choice_multiple

A: multiple answers possible:

money	=>	*I don't have money to buy face masks/Ma haysto lacag aan ku gato maaskiga wajiga*
where_to_get	=>	*I don't know where to get a face mask/Ma aqaan meesha laga helo maaskiga wejiga*
uncomfortable	=>	*Face masks make me uncomfortable/Maaskiga wajiga wuu idhibayaa*
unnecessary	=>	*I don't think that face masks are necessary/Uma maleynayo in maaskiga wajiga loo baahan yahay*

Visible if

Q:	A:
I wear a face mask when going outside/Waxaan xirtaa maaskiga wajiga marka aan dibadda u baxayo	- value => no

Q: I stay at a distance of 1.5-2m from others/Waxaan raacaa qaanuunka kalafogaanshada bulshada 1.5-2 mitirka ah (*)

Type: choice

A: one of the following:

yes	=>	*Yes /Haa*
no	=>	*No /Maya*

Q: When I cough or sneeze, I cover my mouth and nose with a tissue paper/Marka aan qufaco ama hindhiso, waxaan afka iyo sanka ku daboolaa tiish iwm (*)

Type: choice

A: one of the following:

yes	=>	*Yes /Haa*
no	=>	*No /Maya*

Q: I wash my hands using soap and water regularly during the day/ waxaan ku dhaqaa gacmaheyga biyo iyo saabuun si joogto ah maalintii (*)

Type: choice

A: one of the following:

yes	=>	*Yes /Haa*
no	=>	*No /Maya*

Q: I use a hand sanitizer regularly during the day/Waxaan isticmaalaa jeermisdilha gacmaha si joogto ah maalintii (*)

Type: choice

A: one of the following:

yes	=>	*Yes /Haa*
no	=>	*No /Maya*

Q: I stay home when I feel flu-like symptoms/Gurigaan joogaa markaan dareemo wax hargab u eg(*)

Type: choice

A: one of the following:

yes	=>	*Yes /Haa*
no	=>	*No /Maya*

Community preventive measures for coronavirus/Tallaabooyinka looga hortagayo coronavirus ee bulshada

Q: Were you in a meeting or gathering with more than 10 persons during the last 7 days?/Ma ka qaybgashay shir ama kulan in ka badan 10 qof toddobaadkii la soo dhaafay? (*)

Type: choice

A: one of the following:

yes	=>	*Yes /Haa*
no	=>	*No /Maya*

Q: Did you go to a religious gathering during the last 7 days?/Ma aadday kulan diimeed toddobaadkii ugu danbeeyey?(*)

Type: choice

A: one of the following:

yes	=>	*Yes /Haa*
no	=>	*No /Maya*

Q: Did you go to a market in the past 7 days ?/Suuqa ma aadday toddobaadkii ugu tanbeeyey? (*)

Type: choice

A: one of the following:

yes	=>	*Yes /Haa*
no	=>	*No /Maya*

Q: Did you travel in the past 7 days?/ma safartay toddobaadkii ugu danbeeyey? (*)

Type: choice

A: one of the following:

yes_other_provinces	=>	*Yes I traveled to other provinces/Haa waxaan u safray gobollo kale*
yes_outside_country	=>	*Yes I traveled outside the country/Haa waxaan u safray wadanka dibaddiisa*
no_travel	=>	*No travel/Ma safrin*

Q: During the last week, how worried or afraid were you about the health of your loved ones?/Toddobaadkii na dhaafay, sideed u walawalsaneyd ama u cabsaneysey dadkaad jeceshahay? Adigo ku cabbiraya 1= ma walwalsaneyn- ilaa 5= aad ayaan u walwalsanaa (*)

Type: choice_scale

A: 1 = not worried/ma walwalsaneyn to 5 = extremely worried/aad ayaan u walwalsanaa

1	=>	*1*
2	=>	*2*
3	=>	*3*
4	=>	*4*
5	=>	*5*

Q: Would you be willing to take the COVID-19 vaccine when it becomes available?/ Ma doonaysaa inaad qaadato tallaalka COVID-19 marka la helo? (*)

Type: choice

A: one of the following:

yes	=>	*Yes/ Haa*
no	=>	*No /Maya*

Q: If no, why will you refuse the vaccine? (many answers possible)/ Haddii ay maya tahay, maxaad u diidaysaa tallaalka? (jawaabo badan ayaa suuragal ah) (*)

Type: choice_multiple

A: multiple answers possible:

not_effective	=>	*I think the vaccine is not effective/ Waxaan u maleynayaa in tallaalku uusan waxtar lahayn.*
harmful	=>	*I think the vaccine is harmful, I am afraid of side-effects/ Waxaan u maleynayaa in tallaalku dhibaato leeyahay oo waxaan ka baqayaa in uu waxyeelo jirkayga.*
not_needed	=>	*My body is strong, I don't need a vaccine to fight COVID-19/ Jirkayga ayaa xoog badan oo uma baahni tallaal aan kula dagaallamo COVID-19.*
covid_finished	=>	*The COVID-19 pandemic is over in Somalia, no need for a vaccine now/ Cudurka faafa ee COVID-19 waa uu ka dhammaaday Soomaaliya oo hadda tallaal looma baahna.*
other	=>	*Other reasons (please specify)/ Hadday jiraan Sababo kale (fadlan sheeg)*

Visible if

Q:	A:
Would you be willing to take the COVID-19 vaccine when it becomes available?/ Ma doonaysaa inaad qaadato tallaalka COVID-19 marka la helo?	- value => no

Q: Please specify other reasons:

Type: text

A: text input

Visible if

Q:	A:
:input[name="if_no_why_will_you_refuse_the_vaccine_many_options_possible_[other]"]	- checked => 1

Questions related to your personal health/Su'aalo la xiriira caafimaadkaaga shaqsiga ah

Q: Have you experienced any of the following symptoms in the past two weeks? (many answers possible)/Ma isku aragtay calaamadahan soo socdo labadi sitimaan ee lasoodhaafay?(jawaabo badan ayaa bixinkartaa) (*)

Type: choice_multiple

A: multiple answers possible:

fever	=>	*Fever/Qandho/Xummad*
headaches	=>	*Headaches/Madax-xanuun*
sore_throat	=>	*Sore throat/dhuun-xanuun*
loss_taste	=>	*Loss of taste/dhadhan la'aan*
loss_smell	=>	*Loss of smell/Ur la'aan*
stuffy_nose	=>	*Stuffy and/or runny nose/sanka xiran ama diif*
dry_cough	=>	*Dry cough/Qufac qalalan*
productive_cough	=>	*Productive cough/Qufac cantuuf leh*
shortness_breath	=>	*Shortness of breath/Neefsashada oo kugu adkaata*
muscle_pain	=>	*Muscle or body pains/Murqaha ama jir xanuun*
weakness	=>	*General weakness/ Tabardarro*
nausea	=>	*Nausea/Lalabo*
diarrhea	=>	*Diarrhea/shuban*
none	=>	*None of the above symptoms*

Q: Have any of your housemates had any of these symptoms in the last two weeks?/Majiraa qof aad lanooshahay oo qabay astaamaha hergabka labadi sitimaan ee lasoo dhaafay ? (*)

Type: choice

A: one of the following:

yes	=>	*Yes /Haa*
no	=>	*No /Maya*
do_not_know	=>	*Do not know/ Ma garanayo*

Q: Have you been tested for COVID-19?/Ma lagaa baaray COVID-19? (*)

Type: choice

A: one of the following:

yes	=>	*Yes /Haa*
no	=>	*No /Maya*

Q: If yes, what was the result of the test? /Hadday haa tahay, maxuu ahaa natiijada ? (*)

Type: choice

A: one of the following:

positive	=>	*Positive/Bositive*
negative	=>	*Negative/Negative*
do_not_know	=>	*Do not know yet/ Ma garanayo*

Visible if

Q:	A:
Have you been tested for COVID-19?/Ma lagaa baaray COVID-19?	- value => yes

Q: When was this COVID-19 test done? Goorma ayaa Lagaa baaray COVID-19 ? (*)

Type: choice

A: one of the following:

two_weeks	=>	*During the past two weeks/Labadi asbuuc ee lasoo dhaafay gudahooda*
one_month	=>	*More than two weeks, but less than one month ago/Kahor laba isbuuc balse kayar Bil*
more_than_a_month	=>	*I was tested more than a month ago/Waxaa la iga baaray bil kahor*

Visible if

Q:	A:
Have you been tested for COVID-19?/Ma lagaa baaray COVID-19?	- value => yes

Q: According to you, what is the risk that you already had the coronavirus or become infected soon?/Ra'yigaaga, waamaxay khatarta ah inaad qabto coronavirus ama uu kugu dhici karo ? (*)

Type: choice_scale

A: On a scale of 1 to 5/Qiimee hal ilaa shan

1	=>	*1=Very little or no risk/aad uyar ama khatar jirin*
2	=>	*2=Little risk/qatar Yar*
3	=>	*3=Medium risk/khatar dhexdhexaad*
4	=>	*4=High risk/khatar badan*
5	=>	*5=Very high risk/khatar aad ubadan*

Visible if

Q:	A:
0	- :input[name="have_you_been_tested_for_covid_19_"] =>
1	
2	- :input[name="if_yes_what_was_the_result_of_the_test_"] =>

Q: Is there a person in your family who died this year because he or she was infected with COVID-19?? / Ma jiraa qof qoyskaaga ah oo sannadkaan u dhintay COVID-19?? (*)

Type: choice

A: one of the following:

yes	=>	*Yes/ Haa*
no	=>	*No /Maya*
dont_know	=>	*I don't know/ Magaraanyo*

Q: Do you smoke?/Sigaarka ma cabtaa? (*)

Type: choice

A: one of the following:

yes	=>	*Yes /Haa*
no	=>	*No /Maya*

Q: Do you have any of the following chronic/underlying illnesses? (many answers possible)/Maqabtaa cudurada daaimka ah ee soo socda ?(jawaabo badan ayaad dooran kartaa) (*)

Type: choice_multiple

A: multiple answers possible:

Heart disease	=>	*Heart disease/Cudurada wadnaha*
Hypertension	=>	*Hypertension/Dhiig-karka*
Diabetes	=>	*Diabetes/Sokoroow ama macaanka*
Cancer	=>	*Cancer/Kansar*
HIV	=>	*HIV/HIV*
Tuberculosis	=>	*Tuberculosis/Tiibisho*
Asthma	=>	*Asthma/Neefta*
None of the above	=>	*None of the above/ Maqabo*

Q: Do you think that the lockdown measures are necessary to control COVID-19 in Somalia?/ Ma aaminsatahay in xayiraadaha lasoo rogay ay xakamayn karaan cudurka COVID-19 ee gudaha somaalia? (*)

Type: choice

A: one of the following:

yes	=>	*Yes /Haa*
no	=>	*No /Maya*

Q: I fully understand what this study is about, and I consent to participate. All the information I provide can be used by researchers to better understand coronavirus disease in my Country./Waxaan si buuxda u fahamsanahay waxa ay draasaddani ku saabsan tahay, waana oggolahay inaan ka qaybqaato. Dhammaan xogta aan bixinayo waxaa isticmaali kara cilmibaarayaasha si ay si fiican ugu fahmaan cudurka CORONAVIRUS ee ka jira gudaha dalkayga. (*)

Type: checkbox

A: checkbox
