# Supplementary material for: COVID-19 Vaccine Acceptability and Adherence to Preventive Measures in Somalia: Results of an Online Survey
Source: Vaccines (Basel). 2021 May 21;9(6):543. doi: 10.3390/vaccines9060543 (PMC8224389; doi:10.3390/vaccines9060543)
Supplement: Supplementary file 1 [file vaccines-09-00543-s001.zip › File S2.pdf]

APPENDIX 2

OTHER REASONS GIVEN FOR COVID-19 VACCINE HESITANCY

- Because everytime the disease is coming with new face (virus?) and the minimum years to produce vaccine is normally 7-10 years , that is if found
- Because I am not allowed to take medicines
- Because the burden of Covid-19 in Africa is not that much
- Because the contents of the vaccine is haram *[forbidden]* and they made from some haram *[forbidden]* animals such as doofaarka *[pigs]*
- Covid-19 sometime make mutations
- I was already infected with the covid19 and my body is immunized
- I did health check-up 3 months ago and I was not having Covid, so there is no reason to take the vaccine
- I didn't see the vaccine
- I do not trust the vaccine because I did not find what it contains
- I don't like to be the first person on whom the vaccine is tested
- I don't need it
- I just don't want to take the vaccine
- I don't want to take the vaccine because it is made up of some haram *[forbidden]* animals such as pig
- I head from the media the vaccine contain pig fat, so now I am a healthy person how could I inject haram *[forbidden]* into my body !!!
- I surrender myself to God, I will not get problems from covid-19 , may Allah *[God]* protect the nation
- I think I will not need it, because I should have the belief in it before I take any drugs/medicines
- I think the vaccine is not that important to us because we are free from the corona virus
- I trust God
- Infertility
- In Somalia officially there is no covid-19 thus no need for vaccine. if there's effective vaccine, and covid-19 is back people must take the vaccine.
- it is not there (is not available)
- This vaccines have a mission
- Refraining from people who do not need the vaccine, because it may cause new problems like Allergy and etc.
- Still experimental
- The side effects are currently unknown, so No
- The vaccine is not good because is a chemical that can be harmful to yourself
- The vaccine is still experimental, it is not known it's effects in the future
- There is no trusted body who regulates and tests these vaccines, similarly there is no central government to supervise those issues
- This vaccine is good to test in Europe or those of developed countries not in Africa
